# 1-Benzyl-3-[(trimethyl­sil­yl)meth­yl]benzimidazolium chloride monohydrate

**DOI:** 10.1107/S1600536810024128

**Published:** 2010-06-26

**Authors:** Mehmet Akkurt, Ísmail Çelik, Hasan Küçükbay, Nihat Şireci, Orhan Büyükgüngör

**Affiliations:** a Department of Physics, Faculty of Arts and Sciences, Erciyes University, 38039 Kayseri, Turkey; b Department of Physics, Faculty of Arts and Sciences, Cumhuriyet University, 58140 Sivas, Turkey; c Department of Chemistry, Faculty of Arts and Sciences, Ínönü University, 44280 Malatya, Turkey; d Department of Chemistry, Faculty of Arts and Sciences, Adıyaman University, 02040 Adıyaman, Turkey; e Department of Physics, Faculty of Arts and Sciences, Ondokuz Mayıs University, 55139 Samsun, Turkey

## Abstract

The title compound, C_18_H_23_N_2_Si^+^·Cl^−^·H_2_O, was synthesized from 1-[(trimethyl­sil­yl)meth­yl]benzimidazole and benzyl chloride in dimethyl­formamide. The benzimidazole ring system is approximately planar, with a maximum deviation of 0.022 (2) Å, and makes an angle of 74.80 (12)° with the phenyl ring. The crystal packing is stabilized by O—H⋯Cl, C—H⋯Cl, C—H⋯O and C—H⋯π inter­actions between symmetry-related mol­ecules together with π–π stacking inter­actions between the imidazolium and benzene rings [centroid–centroid distance = 3.5690 (15) Å] and between the benzene rings [centroid–centroid distance = 3.7223 (14) Å].

## Related literature

For general background to benzimidazole compounds and for the biological activity of related structures, see: Galal *et al.* (2009 ▶); Huang *et al.* (2006 ▶); Küçükbay & Durmaz (1997 ▶); Küçükbay *et al.* (1995 ▶, 2003 ▶, 2004 ▶, 2010 ▶); Lukevics *et al.* (2001 ▶); Singh & Lown (2000 ▶); Tavman *et al.* (2005 ▶); Turner & Denny (1996 ▶); Williams *et al.* (2002 ▶); Yılmaz & Küçükbay (2009 ▶); Çetinkaya *et al.* (1996 ▶). For similar structures, see: Akkurt *et al.* (2008 ▶, 2010 ▶). 
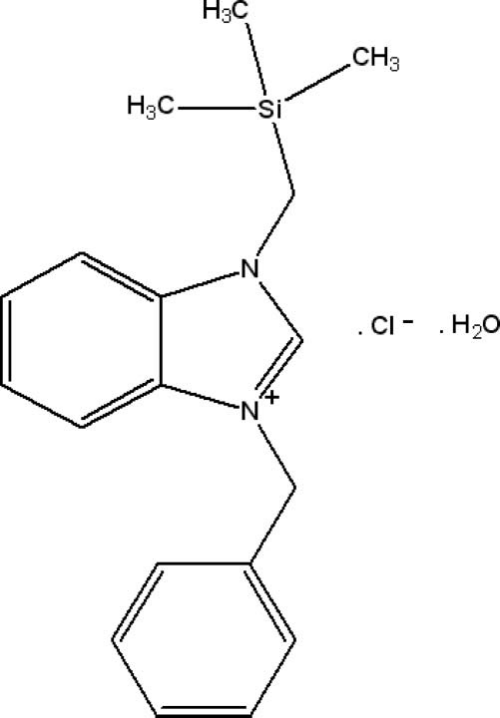


## Experimental

### 

#### Crystal data

C_18_H_23_N_2_Si^+^·Cl^−^·H_2_O
*M* *_r_* = 348.94
Triclinic, 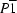

*a* = 9.3592 (7) Å
*b* = 10.9500 (9) Å
*c* = 11.0522 (8) Å
α = 117.594 (6)°
β = 103.295 (6)°
γ = 92.094 (6)°
*V* = 963.39 (15) Å^3^
*Z* = 2
Mo *K*α radiation
μ = 0.27 mm^−1^
*T* = 296 K
0.57 × 0.50 × 0.36 mm

#### Data collection

Stoe IPDS 2 diffractometer
Absorption correction: integration (*X-RED32*; Stoe & Cie, 2002 ▶) *T*_min_ = 0.859, *T*_max_ = 0.909
12149 measured reflections
3987 independent reflections
3241 reflections with *I* > 2σ(*I*)
*R*_int_ = 0.029

#### Refinement

*R*[*F* ^2^ > 2σ(*F* ^2^)] = 0.049
*wR*(*F* ^2^) = 0.135
*S* = 1.07
3987 reflections
211 parameters
H-atom parameters constrained
Δρ_max_ = 0.32 e Å^−3^
Δρ_min_ = −0.32 e Å^−3^

### 

Data collection: *X-AREA* (Stoe & Cie, 2002 ▶); cell refinement: *X-AREA*; data reduction: *X-RED32* (Stoe & Cie, 2002 ▶); program(s) used to solve structure: *SIR97* (Altomare *et al.*, 1999 ▶); program(s) used to refine structure: *SHELXL97* (Sheldrick, 2008 ▶); molecular graphics: *ORTEPIII* (Burnett & Johnson, 1996 ▶), *ORTEP-3 for Windows* (Farrugia, 1997 ▶) and *PLATON* (Spek, 2009 ▶); software used to prepare material for publication: *WinGX* (Farrugia, 1999 ▶).

## Supplementary Material

Crystal structure: contains datablocks global, I. DOI: 10.1107/S1600536810024128/dn2582sup1.cif

Structure factors: contains datablocks I. DOI: 10.1107/S1600536810024128/dn2582Isup2.hkl

Additional supplementary materials:  crystallographic information; 3D view; checkCIF report

## Figures and Tables

**Table 1:** Hydrogen-bond geometry (Å, °) *Cg*3 is the centroid of the C9–C14 ring.

*D*—H⋯*A*	*D*—H	H⋯*A*	*D*⋯*A*	*D*—H⋯*A*
O1—H1*A*⋯Cl1	0.86	2.45	3.257 (2)	157
O1—H1*B*⋯Cl1^i^	0.85	2.45	3.250 (3)	158
C7—H7⋯O1	0.93	2.51	3.170 (3)	128
C8—H8*A*⋯Cl1	0.97	2.81	3.703 (2)	153
C3—H3⋯*Cg*3^ii^	0.93	2.69	3.526 (2)	151

Symmetry codes: (i) 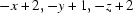; (ii) 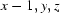.

**Fig. 1.**
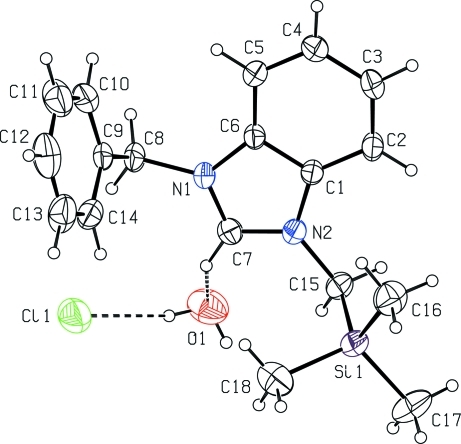
View of the title molecule in the asymmetric unit, with the atom numbering scheme. Displacement ellipsoids for non-H atoms are drawn at the 30% probability level. H atoms are represented as small spheres of arbitrary radii and H bonds are shown as dashed lines.

**Fig. 2.**
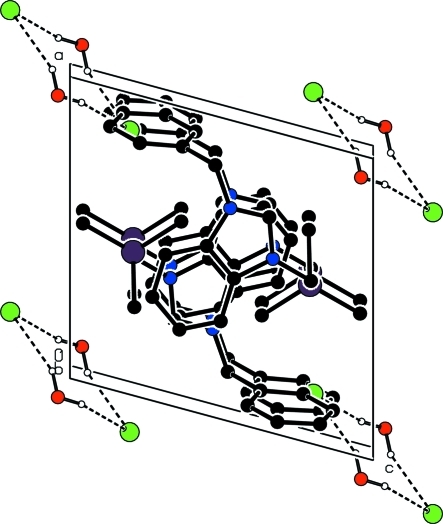
View of the packing and hydrogen bonding interactions of (I) down the b axis. All hydrogen atoms not involved in hydrogen bonding have been omitted for clarity.

## supplementary crystallographic information

### Comment

Although there are different antibacterial and antifungal drugs used in the treatment of bacterial and fungal infections, some of them have undesirable side effects. In addition, some of them become less effective due to the development of resistance to these drugs (Williams *et al.*, 2002). Therefore, many clinically effective antibacterial and antifungal drugs have become less effective due to the development of resistance to these drugs. Since, benzimidazole compounds have been found to have a broad range of pharmacological activity, many research groups as well as our group have been interested in these type of heterocyclic compounds (Singh *et al.*, 2000; Huang *et al.* 2006; Turner & Denny, 1996; Lukevics *et al.*, 2001; Galal *et al.* 2009; Çetinkaya *et al.*, 1996; Küçükbay *et al.*, 1995, 1997, 2003, 2004, 2010; Yılmaz & Küçükbay, 2009; Tavman *et al.*, 2005). In recent years, considerable attention has been given to the synthesis of alkylsilyl substituted benzimidazole derivatives because of their properties in cancer therapy. For example, 1-(3-trimethylsilylpropyl)benzimidazole inhibits carcinoma S-180 tumour growth in dose 1 mg.kg^-1^ by 62% (on ICR mice) (Lukevics *et al.*, 2001). These properties of silylsubstituted benzimidazole compounds, triggered us to synthesis novel trimethylsilyl substituted benzimidazole compounds. The objectives of this study were to synthesize and elucidate the crystal structure of the title compound, 1-benzyl-3-trimethylsilylmethylbenzimidazolium chloride monohydrate, (I).

In the title molecule, (Fig. 1), the benzimidazole ring system (N1/N2/C1–C7) is approximately planar, with maximum deviations of -0.022 (2) Å for C6, -0.018 (2) for C1 and 0.015 (2) for C7. The benzimidazole (N1/N2/C1–C7) and phenyl (C9–C14) systems make an angle of 74.80 (12)°. The values of the geometric parameters in (I) are comparable with those observed for other similar compounds (Akkurt *et al.*, 2008, 2010). The average value of the Si—C bond length is 1.854 (4) Å. The angles around the Si atoms with a distorted tetrahedral geometry rang from 105.86 (16)° to 111.81 (16)°.

The crystal packing of (I) is stabilized by O—H···Cl, C—H···Cl and C—H···π interactions between symmetry-related molecules (Fig. 2 and Table 1), together with π-π stacking interactions between imidazolium and benzene (Table 2).

### Experimental

A mixture of 1-trimethylsilylmethylbenzimidazole (1.02 g, 5 mmol) and benzyl chloride (0.60 cm^3^, 5 mmol) in dimethylformamide (5 ml) was refluxed for 3 h. The mixture was then cooled and the volatiles were removed *in vacuo*. The residue was crystallized from a dimethylformamide/ethanol (1:1). White crystals of the title compound (1.36 g, 82%) were obtained, m.p. 425–426 K; ν_(CN)_ = 1553 cm^-1^. Anal. Found: C 61.64, H 7.19, N 7.93%. Calculated for C_18_H_25_ClN_2_OSi: C 61.96, H 7.22, N 8.03%. ^1^H NMR (δ, DMSO-d_6_): 10.21 (s, 1H, NCHN), 8.11 - 7.59 (m, 4H, C_6_H_4_), 7.56–7.33 (m, 5H, C_6_H_5_), 5.86 (s, 2H, CH_2_ benzyl), 4.30 (s, 2H, CH_2_Si) and 0.14 [s, 9H, (CH_3_)_3_Si]. ^13^C NMR (δ, DMSO-d_6_): 141.6 (NCHN), 134.6, 132.1, 130.8, 129.1, 128.8 and 128.3 (C_6_H_4_), 126.8, 126.5, 114.3 and 113.9 (C_6_H_5_), 49.8 (CH_2_ benzyl), 38.1(CH_2_Si) and -2.5 [(CH_3_)_3_Si].

### Refinement

All H atoms attached to C atoms were fixed geometrically and treated as riding with C—H = 0.93 Å (aromatic), 0.96 Å (methyl) and 0.97 Å (methylene) with U_iso_(H) = 1.2U_eq_(C) or 1.5U_eq_(methyl). H atoms of water molecule were located in difference Fourier maps and included in the subsequent refinement using restraints (O-H= 0.83 (1)Å and H···H= 1.40 (2)Å) with U_iso_(H) = 1.5U_eq_(O).In the last cycles of refinement, they were treated as riding on the O atoms.

### Crystal data

**Table:**

C_18_H_23_N_2_Si^+^·Cl^−^·H_2_O	*Z* = 2
*M**_r_* = 348.94	*F*(000) = 372
Triclinic, *P*1	*D*_x_ = 1.203 Mg m^−^^3^
Hall symbol: -P 1	Mo *K*α radiation, λ = 0.71073 Å
*a* = 9.3592 (7) Å	Cell parameters from 28124 reflections
*b* = 10.9500 (9) Å	θ = 2.1–28.0°
*c* = 11.0522 (8) Å	µ = 0.27 mm^−^^1^
α = 117.594 (6)°	*T* = 296 K
β = 103.295 (6)°	Prism, colourless
γ = 92.094 (6)°	0.57 × 0.50 × 0.36 mm
*V* = 963.39 (15) Å^3^	

### Data collection

**Table:**

Stoe IPDS 2 diffractometer	3987 independent reflections
Radiation source: sealed X-ray tube, 12 x 0.4 mm long-fine focus	3241 reflections with *I* > 2σ(*I*)
plane graphite	*R*_int_ = 0.029
Detector resolution: 6.67 pixels mm^-1^	θ_max_ = 26.5°, θ_min_ = 2.1°
ω scans	*h* = −11→11
Absorption correction: integration (*X-RED32*; Stoe & Cie, 2002)	*k* = −13→12
*T*_min_ = 0.859, *T*_max_ = 0.909	*l* = −13→13
12149 measured reflections	

### Refinement

**Table:**

Refinement on *F*^2^	Primary atom site location: structure-invariant direct methods
Least-squares matrix: full	Secondary atom site location: difference Fourier map
*R*[*F*^2^ > 2σ(*F*^2^)] = 0.049	Hydrogen site location: inferred from neighbouring sites
*wR*(*F*^2^) = 0.135	H-atom parameters constrained
*S* = 1.07	*w* = 1/[σ^2^(*F*_o_^2^) + (0.0637*P*)^2^ + 0.2683*P*] where *P* = (*F*_o_^2^ + 2*F*_c_^2^)/3
3987 reflections	(Δ/σ)_max_ < 0.001
211 parameters	Δρ_max_ = 0.32 e Å^−^^3^
0 restraints	Δρ_min_ = −0.32 e Å^−^^3^

### Special details

**Table:**

Geometry. All esds (except the esd in the dihedral angle between two l.s. planes) are estimated using the full covariance matrix. The cell esds are taken into account individually in the estimation of esds in distances, angles and torsion angles; correlations between esds in cell parameters are only used when they are defined by crystal symmetry. An approximate (isotropic) treatment of cell esds is used for estimating esds involving l.s. planes.
Refinement. Refinement on *F*^2^ for ALL reflections except those flagged by the user for potential systematic errors. Weighted *R*-factors *wR* and all goodnesses of fit *S* are based on *F*^2^, conventional *R*-factors *R* are based on *F*, with *F* set to zero for negative *F*^2^. The observed criterion of *F*^2^ > σ(*F*^2^) is used only for calculating -*R*-factor-obs *etc*. and is not relevant to the choice of reflections for refinement. *R*-factors based on *F*^2^ are statistically about twice as large as those based on *F*, and *R*-factors based on ALL data will be even larger.

### Fractional atomic coordinates and isotropic or equivalent isotropic displacement parameters (Å^2^)

**Table:**

	*x*	*y*	*z*	*U*_iso_*/*U*_eq_	
Si1	0.53328 (7)	0.20172 (7)	0.79168 (6)	0.05705 (19)	
N1	0.72385 (17)	0.36741 (17)	0.52896 (17)	0.0466 (4)	
N2	0.59349 (17)	0.36730 (17)	0.66907 (17)	0.0466 (4)	
C1	0.4923 (2)	0.33506 (19)	0.54087 (19)	0.0428 (4)	
C2	0.3377 (2)	0.3101 (2)	0.4971 (2)	0.0521 (5)	
H2	0.2816	0.3104	0.5566	0.063*	
C3	0.2721 (2)	0.2847 (2)	0.3611 (2)	0.0587 (5)	
H3	0.1688	0.2667	0.3276	0.070*	
C4	0.3560 (2)	0.2853 (3)	0.2717 (2)	0.0592 (5)	
H4	0.3071	0.2682	0.1806	0.071*	
C5	0.5084 (2)	0.3104 (2)	0.3150 (2)	0.0532 (5)	
H5	0.5641	0.3111	0.2555	0.064*	
C6	0.57570 (19)	0.33479 (19)	0.4512 (2)	0.0432 (4)	
C7	0.7293 (2)	0.3855 (2)	0.6574 (2)	0.0505 (5)	
H7	0.8165	0.4079	0.7293	0.061*	
C8	0.8534 (2)	0.3833 (2)	0.4794 (2)	0.0547 (5)	
H8A	0.9413	0.4273	0.5602	0.066*	
H8B	0.8371	0.4442	0.4377	0.066*	
C9	0.8803 (2)	0.2461 (2)	0.3720 (2)	0.0510 (5)	
C10	0.8520 (3)	0.2111 (3)	0.2308 (3)	0.0717 (7)	
H10	0.8171	0.2741	0.2014	0.086*	
C11	0.8748 (3)	0.0831 (4)	0.1329 (3)	0.0935 (10)	
H11	0.8546	0.0600	0.0377	0.112*	
C12	0.9272 (3)	−0.0100 (3)	0.1753 (4)	0.0947 (10)	
H12	0.9404	−0.0970	0.1089	0.114*	
C13	0.9600 (3)	0.0255 (3)	0.3158 (4)	0.0909 (10)	
H13	0.9980	−0.0367	0.3451	0.109*	
C14	0.9372 (3)	0.1527 (3)	0.4141 (3)	0.0700 (7)	
H14	0.9602	0.1760	0.5095	0.084*	
C15	0.5549 (3)	0.3754 (2)	0.7947 (2)	0.0549 (5)	
H15A	0.4625	0.4122	0.8003	0.066*	
H15B	0.6320	0.4406	0.8796	0.066*	
C16	0.3837 (3)	0.0757 (3)	0.6328 (3)	0.0812 (8)	
H16A	0.4063	0.0662	0.5487	0.122*	
H16B	0.2907	0.1090	0.6377	0.122*	
H16C	0.3767	−0.0135	0.6297	0.122*	
C17	0.4849 (4)	0.2374 (4)	0.9569 (3)	0.1001 (10)	
H17A	0.4744	0.1524	0.9619	0.150*	
H17B	0.3928	0.2732	0.9569	0.150*	
H17C	0.5624	0.3050	1.0376	0.150*	
C18	0.7121 (3)	0.1391 (3)	0.7877 (3)	0.0899 (9)	
H18A	0.7028	0.0478	0.7796	0.135*	
H18B	0.7869	0.2023	0.8737	0.135*	
H18C	0.7403	0.1344	0.7076	0.135*	
O1	0.9113 (3)	0.6314 (2)	0.9603 (2)	0.1024 (7)	
H1A	0.9910	0.6000	0.9420	0.154*	
H1B	0.9090	0.6350	1.0380	0.154*	
Cl1	1.14370 (9)	0.44812 (10)	0.80296 (8)	0.0961 (3)	

### Atomic displacement parameters (Å^2^)

**Table:**

	*U*^11^	*U*^22^	*U*^33^	*U*^12^	*U*^13^	*U*^23^
Si1	0.0616 (4)	0.0668 (4)	0.0477 (3)	0.0106 (3)	0.0191 (3)	0.0301 (3)
N1	0.0349 (8)	0.0565 (9)	0.0533 (9)	0.0048 (7)	0.0145 (7)	0.0295 (8)
N2	0.0448 (9)	0.0524 (9)	0.0460 (8)	0.0074 (7)	0.0162 (7)	0.0251 (7)
C1	0.0401 (9)	0.0458 (9)	0.0466 (9)	0.0086 (7)	0.0162 (8)	0.0238 (8)
C2	0.0404 (10)	0.0611 (12)	0.0632 (12)	0.0110 (9)	0.0240 (9)	0.0324 (10)
C3	0.0358 (10)	0.0717 (14)	0.0673 (13)	0.0081 (9)	0.0120 (9)	0.0339 (11)
C4	0.0471 (12)	0.0770 (14)	0.0527 (11)	0.0088 (10)	0.0087 (9)	0.0333 (11)
C5	0.0486 (11)	0.0686 (13)	0.0516 (11)	0.0106 (9)	0.0193 (9)	0.0340 (10)
C6	0.0343 (9)	0.0484 (10)	0.0507 (10)	0.0068 (7)	0.0145 (8)	0.0260 (8)
C7	0.0418 (10)	0.0554 (11)	0.0511 (11)	0.0031 (8)	0.0079 (8)	0.0260 (9)
C8	0.0360 (10)	0.0667 (13)	0.0736 (13)	0.0050 (9)	0.0216 (9)	0.0412 (11)
C9	0.0311 (9)	0.0671 (12)	0.0673 (12)	0.0090 (8)	0.0197 (9)	0.0400 (10)
C10	0.0529 (13)	0.1023 (19)	0.0737 (15)	0.0312 (13)	0.0236 (12)	0.0498 (15)
C11	0.0647 (17)	0.126 (3)	0.0702 (17)	0.0308 (17)	0.0212 (14)	0.0299 (17)
C12	0.0684 (18)	0.0793 (19)	0.120 (3)	0.0195 (15)	0.0432 (18)	0.0266 (18)
C13	0.087 (2)	0.088 (2)	0.146 (3)	0.0402 (16)	0.069 (2)	0.077 (2)
C14	0.0671 (15)	0.0864 (17)	0.0943 (18)	0.0279 (13)	0.0444 (14)	0.0631 (15)
C15	0.0599 (12)	0.0622 (12)	0.0420 (10)	0.0129 (10)	0.0203 (9)	0.0217 (9)
C16	0.0781 (18)	0.0771 (17)	0.0790 (17)	−0.0077 (13)	0.0151 (14)	0.0347 (14)
C17	0.142 (3)	0.111 (2)	0.0703 (17)	0.022 (2)	0.0513 (19)	0.0526 (17)
C18	0.0818 (19)	0.093 (2)	0.088 (2)	0.0247 (16)	0.0135 (16)	0.0423 (17)
O1	0.1114 (17)	0.0974 (15)	0.0822 (13)	0.0288 (13)	0.0144 (12)	0.0355 (12)
Cl1	0.0818 (5)	0.1278 (7)	0.0780 (5)	0.0348 (4)	0.0259 (4)	0.0465 (4)

### Geometric parameters (Å, °)

**Table:**

Si1—C18	1.834 (3)	C9—C10	1.378 (3)
Si1—C17	1.850 (3)	C9—C14	1.383 (3)
Si1—C16	1.852 (3)	C10—C11	1.380 (4)
Si1—C15	1.890 (2)	C10—H10	0.9300
N1—C7	1.328 (3)	C11—C12	1.368 (5)
N1—C6	1.386 (2)	C11—H11	0.9300
N1—C8	1.476 (2)	C12—C13	1.367 (5)
N2—C7	1.324 (2)	C12—H12	0.9300
N2—C1	1.387 (2)	C13—C14	1.376 (4)
N2—C15	1.478 (2)	C13—H13	0.9300
C1—C2	1.389 (3)	C14—H14	0.9300
C1—C6	1.394 (2)	C15—H15A	0.9700
C2—C3	1.374 (3)	C15—H15B	0.9700
C2—H2	0.9300	C16—H16A	0.9600
C3—C4	1.398 (3)	C16—H16B	0.9600
C3—H3	0.9300	C16—H16C	0.9600
C4—C5	1.369 (3)	C17—H17A	0.9600
C4—H4	0.9300	C17—H17B	0.9600
C5—C6	1.384 (3)	C17—H17C	0.9600
C5—H5	0.9300	C18—H18A	0.9600
C7—H7	0.9300	C18—H18B	0.9600
C8—C9	1.497 (3)	C18—H18C	0.9600
C8—H8A	0.9700	O1—H1A	0.8598
C8—H8B	0.9700	O1—H1B	0.8466
			
C18—Si1—C17	111.82 (16)	C14—C9—C8	120.0 (2)
C18—Si1—C16	110.58 (15)	C9—C10—C11	120.5 (3)
C17—Si1—C16	110.93 (16)	C9—C10—H10	119.8
C18—Si1—C15	107.44 (14)	C11—C10—H10	119.8
C17—Si1—C15	105.86 (13)	C12—C11—C10	120.2 (3)
C16—Si1—C15	110.04 (12)	C12—C11—H11	119.9
C7—N1—C6	108.20 (15)	C10—C11—H11	119.9
C7—N1—C8	125.70 (17)	C13—C12—C11	119.7 (3)
C6—N1—C8	126.08 (16)	C13—C12—H12	120.1
C7—N2—C1	108.16 (15)	C11—C12—H12	120.1
C7—N2—C15	126.39 (17)	C12—C13—C14	120.4 (3)
C1—N2—C15	125.43 (16)	C12—C13—H13	119.8
N2—C1—C2	131.97 (17)	C14—C13—H13	119.8
N2—C1—C6	106.56 (16)	C13—C14—C9	120.4 (3)
C2—C1—C6	121.45 (18)	C13—C14—H14	119.8
C3—C2—C1	116.40 (18)	C9—C14—H14	119.8
C3—C2—H2	121.8	N2—C15—Si1	113.64 (13)
C1—C2—H2	121.8	N2—C15—H15A	108.8
C2—C3—C4	122.03 (19)	Si1—C15—H15A	108.8
C2—C3—H3	119.0	N2—C15—H15B	108.8
C4—C3—H3	119.0	Si1—C15—H15B	108.8
C5—C4—C3	121.6 (2)	H15A—C15—H15B	107.7
C5—C4—H4	119.2	Si1—C16—H16A	109.5
C3—C4—H4	119.2	Si1—C16—H16B	109.5
C4—C5—C6	116.92 (18)	H16A—C16—H16B	109.5
C4—C5—H5	121.5	Si1—C16—H16C	109.5
C6—C5—H5	121.5	H16A—C16—H16C	109.5
C5—C6—N1	131.92 (17)	H16B—C16—H16C	109.5
C5—C6—C1	121.62 (17)	Si1—C17—H17A	109.5
N1—C6—C1	106.41 (16)	Si1—C17—H17B	109.5
N2—C7—N1	110.68 (17)	H17A—C17—H17B	109.5
N2—C7—H7	124.7	Si1—C17—H17C	109.5
N1—C7—H7	124.7	H17A—C17—H17C	109.5
N1—C8—C9	112.22 (16)	H17B—C17—H17C	109.5
N1—C8—H8A	109.2	Si1—C18—H18A	109.5
C9—C8—H8A	109.2	Si1—C18—H18B	109.5
N1—C8—H8B	109.2	H18A—C18—H18B	109.5
C9—C8—H8B	109.2	Si1—C18—H18C	109.5
H8A—C8—H8B	107.9	H18A—C18—H18C	109.5
C10—C9—C14	118.7 (2)	H18B—C18—H18C	109.5
C10—C9—C8	121.3 (2)	H1A—O1—H1B	107.0
			
C7—N2—C1—C2	−178.0 (2)	C15—N2—C7—N1	178.44 (17)
C15—N2—C1—C2	3.6 (3)	C6—N1—C7—N2	−0.3 (2)
C7—N2—C1—C6	0.1 (2)	C8—N1—C7—N2	178.18 (18)
C15—N2—C1—C6	−178.27 (17)	C7—N1—C8—C9	109.6 (2)
N2—C1—C2—C3	178.1 (2)	C6—N1—C8—C9	−72.2 (2)
C6—C1—C2—C3	0.2 (3)	N1—C8—C9—C10	108.7 (2)
C1—C2—C3—C4	−0.6 (3)	N1—C8—C9—C14	−72.5 (2)
C2—C3—C4—C5	0.4 (4)	C14—C9—C10—C11	2.3 (4)
C3—C4—C5—C6	0.2 (3)	C8—C9—C10—C11	−178.8 (2)
C4—C5—C6—N1	−177.7 (2)	C9—C10—C11—C12	−0.5 (4)
C4—C5—C6—C1	−0.6 (3)	C10—C11—C12—C13	−1.5 (5)
C7—N1—C6—C5	177.7 (2)	C11—C12—C13—C14	1.7 (5)
C8—N1—C6—C5	−0.7 (3)	C12—C13—C14—C9	0.2 (4)
C7—N1—C6—C1	0.3 (2)	C10—C9—C14—C13	−2.2 (3)
C8—N1—C6—C1	−178.13 (17)	C8—C9—C14—C13	179.0 (2)
N2—C1—C6—C5	−177.97 (18)	C7—N2—C15—Si1	−91.7 (2)
C2—C1—C6—C5	0.4 (3)	C1—N2—C15—Si1	86.4 (2)
N2—C1—C6—N1	−0.2 (2)	C18—Si1—C15—N2	60.61 (19)
C2—C1—C6—N1	178.10 (18)	C17—Si1—C15—N2	−179.77 (18)
C1—N2—C7—N1	0.1 (2)	C16—Si1—C15—N2	−59.84 (19)

### Hydrogen-bond geometry (Å, °)

**Table:**

Cg3 is the centroid of the C9–C14 ring.

**Table:**

*D*—H···*A*	*D*—H	H···*A*	*D*···*A*	*D*—H···*A*
O1—H1A···Cl1	0.86	2.45	3.257 (2)	157
O1—H1B···Cl1^i^	0.85	2.45	3.250 (3)	158
C7—H7···O1	0.93	2.51	3.170 (3)	128
C8—H8A···Cl1	0.97	2.81	3.703 (2)	153
C3—H3···Cg3^ii^	0.93	2.69	3.526 (2)	151

Symmetry codes: (i) −*x*+2, −*y*+1, −*z*+2; (ii) *x*−1, *y*, *z*.

### Table 2 π–π stacking in the title compound (Å, °).

Cg1 is the centroid of the N1, C6, C1, N2, C7 ring and Cg2 is the centroid of C1 to C6 ring. Offset is the angle between the centroid-to-centroid and plane-to-plane vectors.

**Table:**

	Centroid–centroid	plane–plane	offset
Cg1···Cg2^i^	3.5690 (15)	3.430 (1)	16.0
Cg2···Cg2^i^	3.7223 (14)	3.446 (1)	22.2

Symmetry code: (i) 1-x, 1-y, 1-z.
